# Modulation of cortical-subcortical networks in Parkinson’s disease by applied field effects

**DOI:** 10.3389/fnhum.2013.00565

**Published:** 2013-09-13

**Authors:** Christopher W. Hess

**Affiliations:** ^1^Center for Parkinson’s Disease and Other Movement Disorders, Columbia University Medical CenterNY, USA; ^2^University of Florida Center for Movement Disorders and Neurorestoration, GainesvilleFL, USA; ^3^Malcom Randall VA Medical Center, GainesvilleFL, USA

**Keywords:** Parkinson’s disease, transcranial direct current stimulation, transcranial alternating current stimulation, transcranial electrical stimulation, field effects

## Abstract

Studies suggest that endogenous field effects may play a role in neuronal oscillations and communication. Non-invasive transcranial electrical stimulation with low-intensity currents can also have direct effects on the underlying cortex as well as distant network effects. While Parkinson’s disease (PD) is amenable to invasive neuromodulation in the basal ganglia by deep brain stimulation (DBS), techniques of non-invasive neuromodulation like transcranial direct current stimulation (tDCS) and transcranial alternating current stimulation (tACS) are being investigated as possible therapies. tDCS and tACS have the potential to influence the abnormal cortical-subcortical network activity that occurs in PD through sub-threshold changes in cortical excitability or through entrainment or disruption of ongoing rhythmic cortical activity. This may allow for the targeting of specific features of the disease involving abnormal oscillatory activity, as well as the enhancement of potential cortical compensation for basal ganglia dysfunction and modulation of cortical plasticity in neurorehabilitation. However, little is currently known about how cortical stimulation will affect subcortical structures, the size of any effect, and the factors of stimulation that will influence these effects.

## Introduction

Are transcranial direct current stimulation (tDCS) and transcranial alternating current stimulation (tACS) potential treatment modalities for Parkinson’s disease (PD)?

While the foremost treatment for PD continues to be dopaminergic medications, invasive neuromodulation through deep brain stimulation (DBS) has become a mainstay of therapy in selected patients (Okun, 2012). As in a variety of other neurological disorders (Rothwell, 2012; Schulz et al., 2013), techniques of non-invasive neuromodulation are also being investigated as possible treatment options for PD (Cantello, 2002; Fregni et al., 2005; Edwards et al., 2008; Wu et al., 2008; Lefaucheur, 2009). However, PD is relatively unique amongst these diseases in that the targeted network involves cortical-subcortical activity rather than just cortical activity. This mini-review discusses the use of non-invasive applied electrical fields in PD and considers their potential to influence cortical oscillations and modulate dysfunctional cortical-subcortical networks through the application of weak exogenous fields.

## A rationale for transcranial electrical stimulation in the treatment of Parkinson’s disease

Although classically considered a disease of the basal ganglia, functional imaging and EEG studies have shown altered cortical activity in the supplementary motor area (SMA), dorsolateral prefrontal cortex (DLPFC), and primary motor cortex (M1) in patients with PD (Priori and Lefaucheur, 2007). Moreover, synchronization of oscillatory activity in the motor cortices at specific frequencies is believed to be important in normal motor control (Joundi et al., 2012), and excessive oscillatory activity and abnormal synchronization in the beta band may play a role in the manifestation of PD symptoms (Eusebio and Brown, 2009; Shimamoto et al., 2013). Though the relationship between beta oscillations and PD remains poorly understood, it is rooted in the observations of enhanced beta frequency oscillations in the basal ganglia in PD that are correlated with clinical symptoms and improvement from dopaminergic medications, as well as a worsening of motor symptoms that can be seen by inducing beta oscillations in subthalamic nucleus (STN) using DBS (Stein and Bar-Gad, 2013). Recent studies have suggested that this activity might be cortical in origin, with hyperactivity of the STN occurring secondary to abnormal motor cortical activity transmitted via the hyperdirect pathway (Litvak et al., 2011; Crowell et al., 2012). Further, the clinical efficacy of STN DBS in PD may involve antidromic effects upon the motor cortex (Gradinaru et al., 2009), and high frequency DBS has been shown to decrease beta frequency power in the cortical origin of the hyperdirect pathway that is coherent with beta frequency activity in the STN (Whitmer et al., 2012).

**Figure 1:**
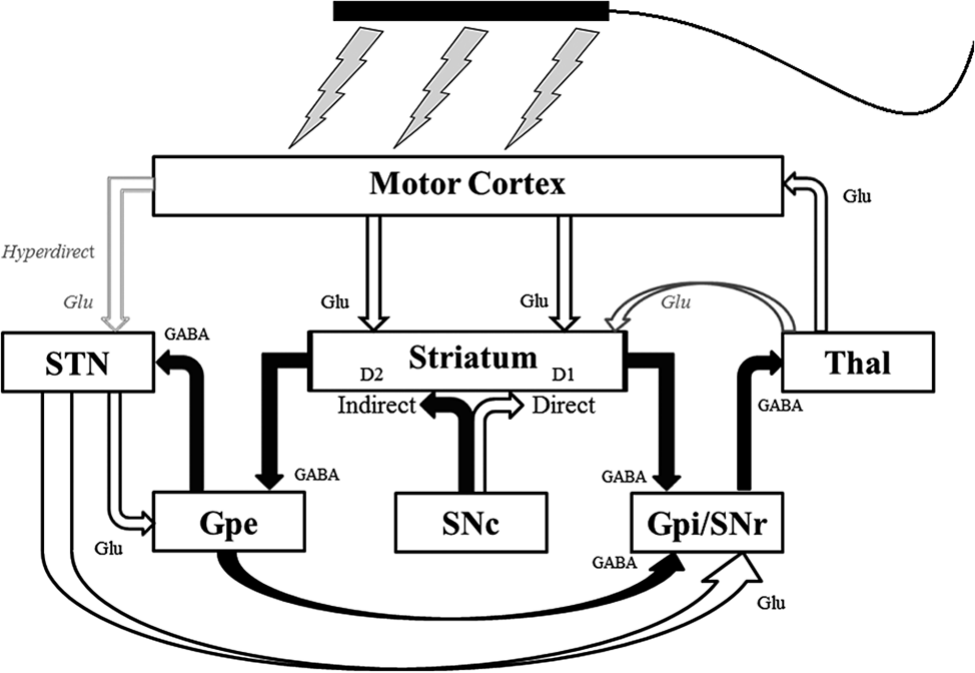
**Schematic of the pathways of the basal ganglia-thalamocortical network that non-invasive transcranial electrical stimulation could potentially influence**. Unfilled arrows are excitatory connections. Filled arrows are inhibitory connections. D1 = D1 dopaminergic receptors; D2 = D2 dopaminergic receptors; GABA = *γ*-Aminobutyric acid (GABA)-ergic; Glu = glutaminergic; Gpe = external segment of the globus pallidus; Gpi = internal segment of the globus pallidus; SNc = substantia nigra pars compacta; SNr = substantia nigra pars reticulata; STN = subthalamic nucleus; Thal = thalamus. Modified with permission from Hess et al. (2013).

Techniques of non-invasive neuromodulation such as tDCS and tACS have the potential to influence the abnormal cortical-subcortical network activity that occurs in PD (Figure 1). tDCS is believed to exert its primary influence on the CNS through extracellular field effects upon membrane potentials (Paulus, 2011) in both a site and polarity specific manner (Zaghi et al., 2010). In general, anodal stimulation increases cortical excitability, while cathodal stimulation decreases it (Nitsche and Paulus, 2000, 2001). Longer-acting effects are likely mediated by separate polarity-specific effects on synaptic plasticity (Liebetanz et al., 2002; Fritsch et al., 2010; Stagg and Nitsche, 2011). In addition to modulating local cortical excitability, neuroimaging studies have demonstrated the ability of tDCS to influence regional cerebral blood flow (rCBF) and resting-state functional connectivity in distant but anatomically and/or functionally connected areas (Lang et al., 2005; Zaghi et al., 2010; Keeser et al., 2011). Thus tDCS could potentially ameliorate PD symptomatology through the induction of sub-threshold changes in excitability in key cortical nodes of the basal ganglia-thalamocortical pathway or produce long-term effects on synaptic plasticity. The putative mechanism of action of tACS is less clear, but may include entrainment or disruption of ongoing rhythmic cortical activity (Zaghi et al., 2010). This could make tACS an ideal modality to interfere with the abnormal oscillatory activity that occurs in the basal ganglia-thalamocortical network in PD (Brittain et al., 2013).

## tDCS in Parkinson’s disease

The study of the therapeutic potential of tDCS in PD is still largely preliminary, yet with some promising findings (Rothwell, 2012). In animal models, cathodal tDCS increased extracellular dopamine levels as measured by striatal microdialysis in healthy rats (Tanaka et al., 2013), and anodal tDCS of M1 improved motor function in the 6-hydroxydopamine rat model of PD (Li et al., 2011). In patients with PD, a single session of sham-controlled anodal tDCS of M1 yielded improvements in motor function that were different from sham stimulation (Fregni et al., 2006). One randomized, double-blind, sham-controlled trial examined the effects of tDCS in PD (Benninger et al., 2010). Subjects underwent eight sessions of tDCS (*n* = 13) or sham stimulation (*n* = 12) while on medication, with stimulation in the tDCS group alternating between the premotor/motor area and prefrontal cortex stimulation. tDCS decreased walking time (the primary outcome) compared to sham one day after stimulation, but only when tested off medications and after exclusion of an outlier in the sham group. Though motor Unified Parkinson’s Disease Rating Scale (UPDRS) and reaction time changes did not differ between groups, upper extremity bradykinesia was significantly improved at all evaluations periods up to three months after stimulation. In addition to motor symptoms, a wide variety of non-motor symptoms occur in PD that are not responsive to levodopa therapy and could potentially be treated with tDCS (Wu et al., 2008). Left DLPFC anodal stimulation has been shown to improve working memory in PD patients (Boggio et al., 2006), and anodal DLPFC tDCS improved phonemic fluency and enhanced fMRI measures of functional connectivity in verbal fluency related networks (Pereira et al., 2013).

## tACS in Parkinson’s disease

As in tDCS, the literature related to PD using tACS is sparse, though intriguing. tACS at varying frequencies was shown to modulate the rate of force development and peak force in handgrip response to a go/no-go task (Joundi et al., 2012), and tACS administered in the beta band (20 Hz) to M1 slowed voluntary movement speed in healthy subjects (Pogosyan et al., 2009). While in one study (Shill et al., 2011) tACS over the forehead and mastoids did not significantly influence off medication UPDRS scores in early PD patients, a recent study achieved a reduction in tremor amplitude of up to 53% using tACS over the contralateral M1 in patients with tremor-dominant PD (Brittain et al., 2013). In this study, tACS at tremor frequency, double tremor frequency, and sham (30 seconds of stimulation) was applied in random order to 12 patients over the transcranial magnetic simulation (TMS)-demonstrated motor hot spot for the muscles most involved with tremor. Stimulation was first allowed to drift in and out of phase with tremor to determine the most effective phase relationship in reducing tremor. In a subset of five patients, stimulation at tremor frequency was given for 30 seconds, during which tremor frequency and the phase relationship between tremor and stimulation was monitored and adjusted in real time. Resting tremor amplitude was reduced by an average of 42%. Further, stimulation did not interfere with performance on pegboard tasks, suggesting that normal motor activity would likely not be affected.

## Conclusions

The application of non-invasive applied electric fields provides a potential window through which the dysfunctional subcortical-cortical networks in PD can be accessed and influenced. However, it remains largely speculative how cortical stimulation will affect subcortical structures, what the effect size will be, and the factors of stimulation that will influence these effects. Given the progressive neurodegenerative nature of the disease and the increasing recognition of the full range of symptoms associated with it, the utility of these techniques may be more as adjuncts to other therapies (Chen, 2010). This being said, the ease of use and low cost of transcranial electrical stimulation makes its development for possible clinical uses appealing (Brunoni et al., 2013). In addition to modulating basal ganglia-thalamocortical network activity, tDCS and tACs may also be useful in promoting cortical compensation for basal ganglia network dysfunction (Fregni et al., 2006) and amplifying cortical plasticity during physical therapy and neurorehabilitation (Chen, 2010; Block and Celnik, 2012). tDCS has also already shown promise in the treatment of non-motor cognitive symptoms in PD, for which current therapies are quite limited when compared to therapies for motor symptoms (Boggio et al., 2006; Wu et al., 2008; Pereira et al., 2013).

A better understanding of the mechanisms by which non-invasive electrical stimulation affect neural networks would likely streamline the discovery of any potential therapeutic applications in PD. Yet as we have seen with DBS in PD, our mechanistic understanding can sometimes lag behind successful therapeutic implementation. Further studies will help to clarify factors such as the optimal montages, sites, and intervals of stimulation (Paulus, 2011), as well as potential interactions with levodopa and other pharmacologic agents (Chaieb et al., 2012).

## Conflict of interest statement

The authors declare that the research was conducted in the absence of any commercial or financial relationships that could be construed as a potential conflict of interest.
